# *Sergentomyia khawi*: a potential vector for *Leishmania* and *Trypanosoma* parasites affecting humans and animals and insecticide resistance status in endemic areas of Songkhla, southern Thailand

**DOI:** 10.1186/s13071-024-06440-0

**Published:** 2024-08-21

**Authors:** Atchara Phumee, Nataya Sutthanont, Suwalak Chitcharoen, Vorthon Sawaswong, Rungfar Boonserm, Pattama Ayuyoe, Ana Cantos-Barreda, Padet Siriyasatien

**Affiliations:** 1 Department of Medical Technology, School of Allied Health Sciences, Walailak University, Nakhon Si Thammarat, Thailand; 2 Excellent Center for Dengue and Community Public Health (EC for DACH), Walailak University, Nakhon Si Thammarat, Thailand; 3 Department of Medical Entomology, Faculty of Tropical Medicine, Mahidol University, Bangkok, Thailand; 4 Department of Microbiology, Faculty of Medicine, Khon Kaen University, Khon Kaen, Thailand; 5 Department of Biochemistry, Faculty of Science, Mahidol University, Bangkok, Thailand; 6 Center of Excellence in Vector Biology and Vector Borne Diseases, Department of Parasitology, Faculty of Medicine, Chulalongkorn University, Bangkok, Thailand; 7 Department of Parasitology, King Chulalongkorn Memorial Hospital, Thai Red Cross Society, Bangkok, Thailand; 8 Department of Biochemistry and Molecular Biology-A, Faculty of Veterinary Medicine, Regional Campus of International Excellence “Campus Mare Nostrum”, University of Murcia, Murcia, Espinardo Spain; 9 Animal Health Department, University of Murcia, Murcia, Espinardo Spain

**Keywords:** *Sergentomyia khawi*, *Leishmania martiniquensis*, *L. orientalis*, *Trypanosoma* sp., Insecticide resistance status

## Abstract

**Background:**

Sand flies serve as crucial vectors in various medical and veterinary diseases. Sand fly-borne diseases pose a significant public health burden globally, as the causative agents can infect a diverse range of hosts, leading to severe consequences such as leishmaniasis and sand fly fever. Additionally, the widespread use of insecticides for agricultural purposes and mosquito control is not specifically targeted at sand flies, potentially leading to resistance development. We investigated sand fly species, their potential role as vectors of various parasitic agents, and insecticide resistance in the endemic regions of Natawi and Sadao districts in Songkhla, Thailand.

**Methods:**

Sand flies were collected using CDC light traps. The collected sand flies were then identified to species level using molecular techniques. Subsequent analyses included the detection of pathogens and the identification of pyrethroid resistance mutations within the voltage-sensitive sodium channel (*Vgsc*) domain IIS6 gene, followed by sequence analysis.

**Results:**

The study identified nine sand fly species belonging to the genera *Phlebotomus* and *Sergentomyia*. The DNA of *Sergentomyia khawi* was the only species found to test positive for one sample of *Leishmania orientalis* in Sadao district. This finding represents the first detection of *L. orientalis* in Thailand. Moreover, three samples of *Leishmania martiniquensis* and four samples of *Trypanosoma* sp. were found in the Natawi district. No I1011M, L1014F/S, V1016G, or F1020S mutations were detected in *Vgsc* gene.

**Conclusions:**

The results of this study provide valuable information on sand fly species and the continuous circulation of *Leishmania* spp. and *Trypanosoma* spp. in Songkhla, southern Thailand. Moreover, the development of geo-spatial information on vectors, parasites, and insecticide resistance in sand flies has the potential to provide well-informed risk assessments and evidence-based guidance for targeted vector control in Thailand. These results can serve as a foundation for integrating the One Health approach, which is crucial for disease control, considering the diverse ecological interactions among human and/or animal reservoir hosts, parasites, and sand fly vectors.

**Graphic Abstract:**

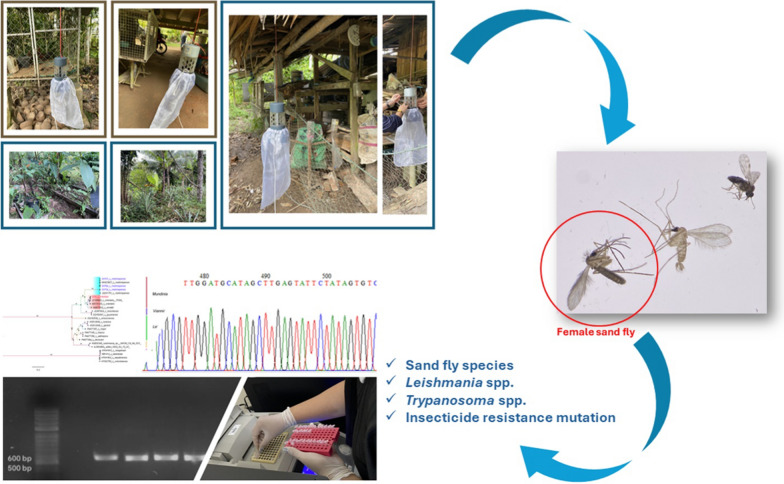

**Supplementary Information:**

The online version contains supplementary material available at 10.1186/s13071-024-06440-0.

## Background

Sand flies are tiny blood-feeding (hematophagous) insects belonging to the order Diptera, family Psychodidae, and subfamily Phlebotominae [1]. These diminutive, hairy insects have slender bodies and up-held V-shaped wings when resting, distinguishing them from other small insects [2]. Approximately 800 species of sand flies have been identified worldwide. According to the widely accepted classification, there are three genera in the New World, *Lutzomyia*, *Psychodopygus*, and *Nyssomyia*, and eight genera in the Old World, *Phlebotomus*, *Sergentomyia*, *Grassomyia*, *Spelaeomyia*, *Idiophlebotomus*, *Parvidens*, *Spelaophlebotomus*, and *Chinius* [3–5]. Among them, *Phlebotomus* (*Ph.*) and *Sergentomyia* (*Se.*) are the most prevalent. At least 39 species of *Phlebotomus* are known to feed on humans [6]. The genus *Sergentomyia* is recognized for possessing the greatest known diversity among sand flies [7]. These sand flies play a crucial role as vectors for various established, emerging, and re-emerging infectious diseases, including leishmaniasis and sand fly-borne phleboviruses, impacting both human and animal health [8]. The World Health Organization (WHO) estimates an annual incidence of 700,000 to 1,000,000 patients and 20,000 to 30,000 deaths due to leishmaniasis [9]. This complexity in *Leishmania* (*L.*) parasite transmission underscores the necessity for the One Health approach, which becomes imperative for controlling leishmaniasis given the intricate ecological relationships among human and/or animal reservoir hosts, parasites, and sand fly vectors [10]. In Thailand, autochthonous leishmaniasis is caused by several species: *Leishmania martiniquensis* [11, 12], *L. orientalis* [13, 14], *L. donovani* [15], and *L. infantum* [16]. The reports have identified cases in the central, northern, and southern regions of the country. In 2015, WHO declared Thailand, previously considered free from the disease, as an endemic area for cutaneous leishmaniasis [17]. Currently, the number of autochthonous leishmaniasis cases is significantly increasing. Furthermore, sand flies in Thailand have been found to harbor *L. martiniquensis* DNA, including species like *Sergentomyia* (*Neophlebotomus*) *gammae*, *Se. khawi*, and *Se.* (*Parrotomyia*) *barraudi*. Additionally, *L. martiniquensis* DNA was detected in rats (*Rattus rattus*) using *ITS1*-PCR in southern Thailand [18, 19]. Trypanosomiasis, a zoonotic disease with diverse symptoms, infects various animals in Asia, including cattle [20], rats [21, 22], deer [23], and humans [24]. The most common species found are *Trypanosoma* (*T.*) *evansi* and *T. lewisi* [21, 25]. While tsetse flies are well-known trypanosome vectors, these blood-sucking insects are not present in Asia. Here, transmission likely occurs through various hematophagous arthropods like mosquitoes, leeches, and kissing bugs [26]. Interestingly, sand flies are believed to potentially transmit trypanosomes to bats [27], snakes [28], and lizards [29]. Significantly, Phumee et al. (2017) detected the first presence of *Trypanosoma* sp. DNA (potentially indicative of a new *Trypanosoma* species) in a *Phlebotomus stantoni* sand fly from southern Thailand [30]. Presently, Thailand lacks comprehensive information regarding the diversity of sand flies and associated pathogens. Preventing sand fly-borne diseases relies significantly on vector control, which aims to reduce sand fly populations and interrupt disease transmission. However, no prior data exist on the insecticide susceptibility and resistance of sand flies in Thailand. Pyrethroids, the main insecticides used for controlling adult and immature mosquitoes, might indirectly combat sand flies [31]. The major mechanisms of pyrethroid resistance in insects involve knockdown resistance mutations (*kdr*) within the para voltage-gated sodium channel gene (*Vgsc*) in nerve cells [32]. The widespread use of insecticides for vector control can lead to increased resistance among sand flies. Understanding the patterns and distributions of *kdr* mutations in sand flies highlights the necessity for an effective vector control program. Therefore, our study aims to survey sand fly species composition, screen for sand fly-borne pathogens, and evaluate insecticide resistance at the *Vgsc* domain IIS6 region using molecular diagnostic tools in endemic areas of Songkhla, southern Thailand. These data are essential for implementing effective vector control strategies to prevent the transmission of sand fly-borne pathogens and safeguard public health.

## Methods

### Study areas and sample collection

Sand fly surveillance was conducted in January 2023 within two districts of Songkhla province, Natawi (6°39′28″N, 100°42′49″E) and Sadao (6°38′19″N, 100°25′26″E). Detailed GPS coordinates and brief descriptions of each location are provided. The surveillance team employed CDC miniature light trap, designed by the US Centers for Disease Control and equipped with 25W bulb and ultraviolet (UV) light, to capture sand flies. Six traps were strategically positioned at various indoor and outdoor locations at each site, including areas under a Thai house, animal shed, and chicken coop; around termite mounds; under coconut trees; and within shrubbery. The traps were set approximately 0.5 to 1.5 m above the ground and operated from 6:00 p.m. to 6:00 a.m. the following morning. Collections at each site spanned an average of 3 nights before being transported to the laboratory for further processing. Insects collected from the light traps were anesthetized at − 20 °C for 30 min. All sand flies were morphologically differentiated according to their gender under a stereomicroscope (Olympus, Japan).

### DNA extraction

Each individual sand fly was lysed in 200 µl lysis buffer supplemented with 20 µl proteinase K. The samples were then homogenized using a sterile plastic pestle. Genomic DNA extraction was performed utilizing commercially available Invisorb Spin Tissue Mini Kit (STRATEC molecular GmbH, Germany) following the manufacturer’s protocols. Subsequently, the DNA was eluted in 50 µl elution buffer. For long-term storage, the extracted DNA was maintained at − 80 °C.

### Molecular identification of sand fly species

For sand fly DNA species identification, we employed primers CB3-PDR (5’ CAY-ATT-CAACCW-GAA-TGA-TA 3’) and N1N-PDR (5’ GGT-AYW-TTG-CCTCGA-WTT-CGW-TAT-GA 3’) to amplify the cytochrome B (*CytB*) gene, resulting in a 500-bp amplicon, adhering to a methodology previously described by Ready et al. (1997) [33]. In brief, the PCR reaction mixture, with a total volume of 25 µl, included 12.5 µl 2X green PCR master mix direct-load (Biotechrabbit, Germany), 0.4 µl of each primer (10 pmol/μl), 8.7 µl deionized distilled water (ddH_2_O), and 3 µl DNA template. The ddH_2_O was used as a negative control. The PCR reaction program protocol was executed according to the following steps: initial denaturation at 94 °C for 3 min; followed by five cycles consisting of denaturation at 94 °C for 1 min, annealing at 40 °C for 1 min, and extension at 68 °C for 1 min; subsequently, 35 cycles of denaturation at 94 °C for 1 min, annealing at 44 °C for 1 min, and extension at 68 °C for 1 min; finally, a concluding extension step at 68 °C for 10 min.

### Detection of *Leishmania* and *Trypanosoma* parasite DNA

PCR amplification was annealed specifically to the nuclear ribosomal internal transcribed spacer 1 (*ITS1*) region of *Leishmania* parasites and the small subunit ribosomal ribonucleic acid (*SSU rRNA*) gene of *Trypanosoma* parasites. For *Leishmania* spp., the reactions were performed using primers LeR: 5′ CCA-AGT-CAT-CCA-TCG-CGA-CAC-G 3′ and LeF: 5′ TCC-GCCCGA-AAG-TTC-ACC-GAT-A 3′, targeting a fragment of approximately 370 bp [34]. For *Trypanosoma* spp., a set of primers TRY927-F: 5′ AGA-AAC-ACG-GGA-G 3′ and TRY927-R: 5′ CTA-CTG-GGC-AGC-TTG-GA 3′ was applied to amplify approximately 900 bp as described by Noyes et al. (1999) [35]. PCR reactions were prepared in a total volume of 25 µl using green hot start PCR master mix direct load (Biotechrabbit, Germany) in a PCR mastercycler (Eppendorf, Germany). The reaction conditions included an initial denaturation step at 94 °C for 4 min, followed by 40 cycles of denaturation at 94 °C for 1 min, annealing at 65 °C for 1 min for the *ITS1* gene or 51.7 °C for the *SSU rDNA* gene, and extension at 72 °C for 1 min. Subsequently, a final extension step was conducted at 72 °C for 7 min. The resulting PCR products underwent analysis by electrophoresis on a 1.5% agarose gel for 40 min at 100 V and were then visualized using Quantity One Quantification Analysis Software Version 4.5.2 (Bio-Rad, USA).

### Identification of mutations in the voltage-gated sodium channel (*Vgsc*) region

The conserved primers Vssc8F (5′ AAT-GTG-GGA-TTG-CAT-GCT-GG 3′) and Vssc1bR (5′ CGT-ATC-ATT-GTC-TGC-AGT-TGG-T 3′) [36] were designed to amplify a genomic DNA fragment from the *Vgsc* domain II, segment 6. These primers were used to monitor the presence and frequency of the *kdr* mutations at codon 1011, 1014, 1016, and 1020, specifically targeting mutations I1011M, L1014F/S, V1016G, and F1020S in sand flies. Each amplification was conducted in a 25 μl PCR reaction mixture, which comprised 2X green PCR master mix direct load (Biotechrabbit, Germany), specific primers, ddH_2_O, and the DNA template. The thermocycling conditions were set as follows: an initial denaturation at 95 °C for 5 min, followed by 35 cycles of 96 °C for 30 s, 56 °C for 30 s, and 72 °C for 30 s, concluding with a final extension at 72 °C for 5 min. The complete *Vgsc* sequence of *Musca domestica* (house fly) (accession no. X96668) and partial sequences of *Phlebotomus argentipes* (accession nos. KY114616-KY114619) were obtained from GenBank.

### Gel purification and sequencing

The corresponding bands from the gels, which exhibited clear, single bands, were purified using ExoSAP-IT (Biotechrabbit, Germany), following the manufacturer’s instructions. In cases where positive bands displayed multiple bands on gel electrophoresis, they were excised from the gels and purified using the agarose gel DNA purification kit Invisorb Fragment CleanUp (STRATEC molecular GmbH, Germany), following the manufacturer’s instructions. Subsequently, the purified DNA samples were sent for direct DNA sequencing to Macrogen, Inc. (Macrogen Inc., South Korea).

### DNA cloning and sequencing

For faint or multiple bands on gel electrophoresis, PCR amplicons were ligated into pGEM-T Easy Vector (Promega, USA). The ligation reaction mixture consisted of 5 µl of 2X Rapid ligation buffer, 3 µl of PCR products, 1 µl of pGEM-T Easy Vector, and 1 µl of ddH_2_O. Subsequently, the ligated vector was transformed into DH5α competent cells, and chimeric plasmids were screened using the blue-white colony selection system. Suspected positive colonies were cultured and utilized for further plasmid DNA extraction, employing the Invisorb Spin Plasmid Mini kit (STRATEC Molecular GmbH, Germany), following the manufacturer’s instructions. Purified plasmids were then forwarded to Macrogen, Inc. (South Korea) for Sanger sequencing service using the universal forward T7 primer.

### Sequences and phylogenetic analysis

Nucleotide sequences were analyzed using BioEdit Sequence Alignment Editor, version 7.0.9.0; the consensus sequences were compared with available sequence data in the GenBank by BLAST search (available at http://www.ncbi.nlm.gov/BLAST). This tool searches nucleotide databases using % nucleotide queries and identity. Phylogenetic trees were generated using the maximum-likelihood method with IQ-TREE on the IQ-TREE web server (http://iqtree.cibiv.univie.ac.at/) with 1000 ultrafast bootstrap replicates. The best fit model of substitution was identified using the auto function on the IQ-TREE web server (http://iqtree.cibiv.univie.ac.at/). The phylogenetic tree is finally viewed and edited with FigTree version 1.4.4 (http://tree.bio.ed.ac.uk/software/figtree/).

### Statistical analyses

Descriptive statistics were used to determine the estimated prevalence, expressed as a percentage. The prevalence calculation employed a formula established from a pilot study and a previous publication by our team. A 95% confidence interval was used. In simpler terms, the prevalence was calculated by dividing the number of sand flies collected during the survey by the total number of sand fly samples. All statistical analyses were conducted using Microsoft Excel 2019 (Microsoft Corp., USA).

## Results

### Molecular identification of sand fly species

**Fig. 1:**
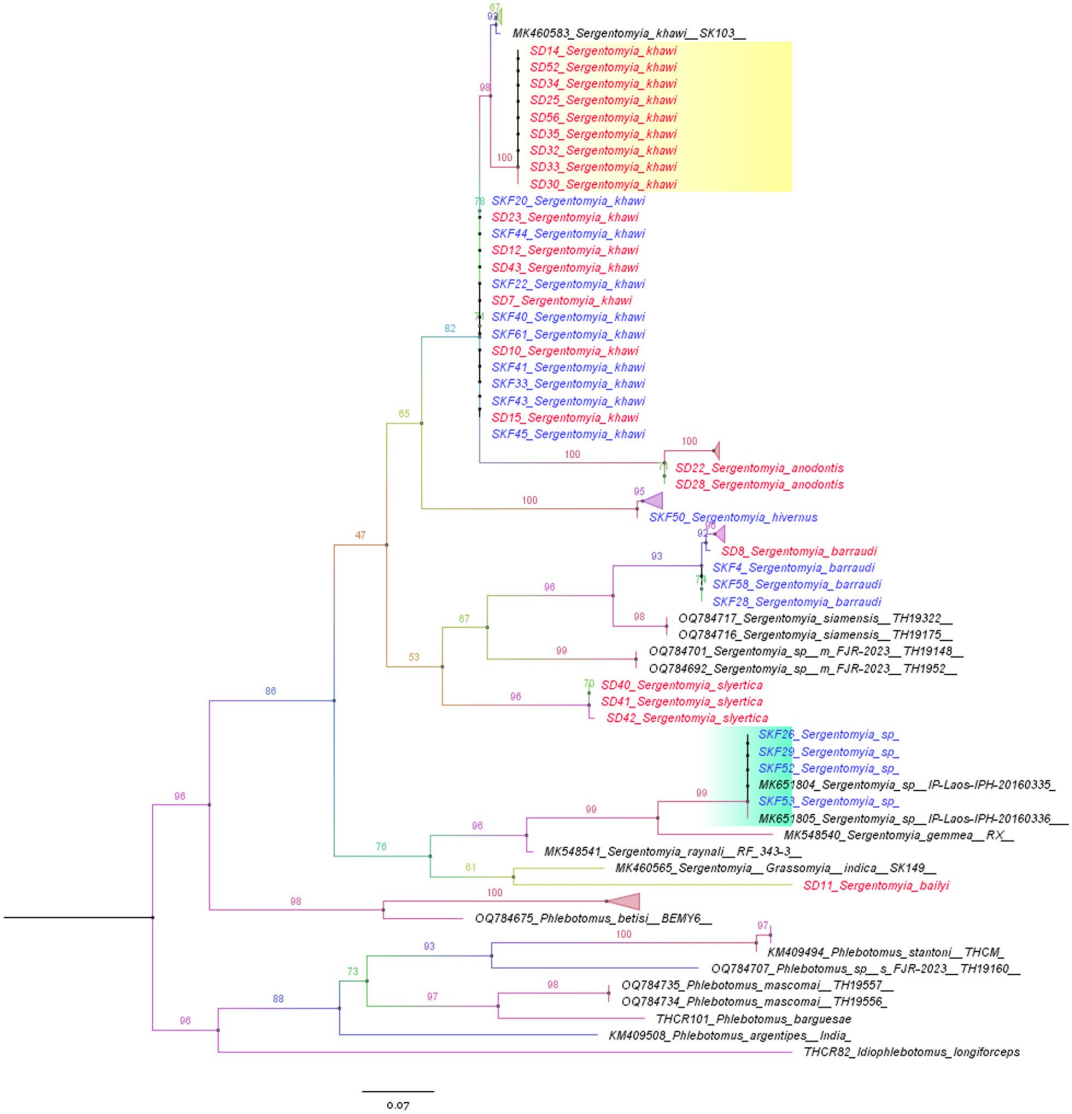
The phylogenetic tree of *CytB* gene sequences among various sand fly species. The tree was constructed using IQ-TREE with maximum-likelihood bootstrap support (1000 replicates). The best-fit substitution model was determined using the auto function on the IQ-TREE web platform. Sequences from the Natawi and Sadao districts are differentiated by blue and red colors, respectively

A total of 121 female sand flies were collected for this study, with 62 (51.2%) samples obtained from Natawi district and 59 (48.8%) from Sadao district. Molecular identification revealed these sand flies belonged to two genera and nine species. In the Natawi, the identified species included *Phlebotomus stantoni*, *Sergentomyia barraudi*, *Se. khawi*, *Se. hivernus*, and *Sergentomyia* sp. Sadao district had *Phlebotomus betisi*, *Sergentomyia barraudi*, *Se. khawi*, *Se. bailyi*, *Se. anodontis*, and *Se. slyertica*. The composition of sand fly fauna exhibited distinctive characteristics in each district. *Phlebotomus stantoni*, *Se. hivernus*, and *Sergentomyia* sp. were exclusively found in the Natawi district, whereas *Ph. betisi*, *Se. bailyi*, *Se. anodontis*, and *Se. slyertica* were identified solely in the Sadoa district. Notably, *Se. khawi* was the most prevalent species in both districts, accounting for 40 out of 62 samples in Natawi and 35 out of 59 samples in Sadao (see Additional file 1). The phylogenetic tree constructed based on the *CytB* gene of sand fly species revealed a well-supported clade, providing clear insights into the relationships among various sand fly species, including *Se. khawi*, *Se. anodontis*, *Se. hivernus*, *Se. barraudi*, *Se. slyertica*, *Sergentomyia sp.*, *Se. bailyi*, *Ph. stantoni*, and *Ph. betisi*. Interestingly, four specimens of *Sergentomyia* sp. from the Natawi clustered with sand flies previously recorded in the Lao People’s Democratic Republic (Lao PDR), specifically referenced as IP-Laos-IPH-20160335 (accession no. MK651804) and IP-Laos-IPH-20160336 (accession no. MK651805). The analysis of *Se. khawi* from both the Natawi and Sadao districts revealed significant genetic diversity (0.5–3%). Notably, a subset of *Se. khawi* from the Sadao formed a distinct sister clade separate from the major *Se. khawi* clade (Fig. 1).

### Molecular detection of *Leishmania* and *Trypanosoma* parasites in sand flies

**Fig. 2:**
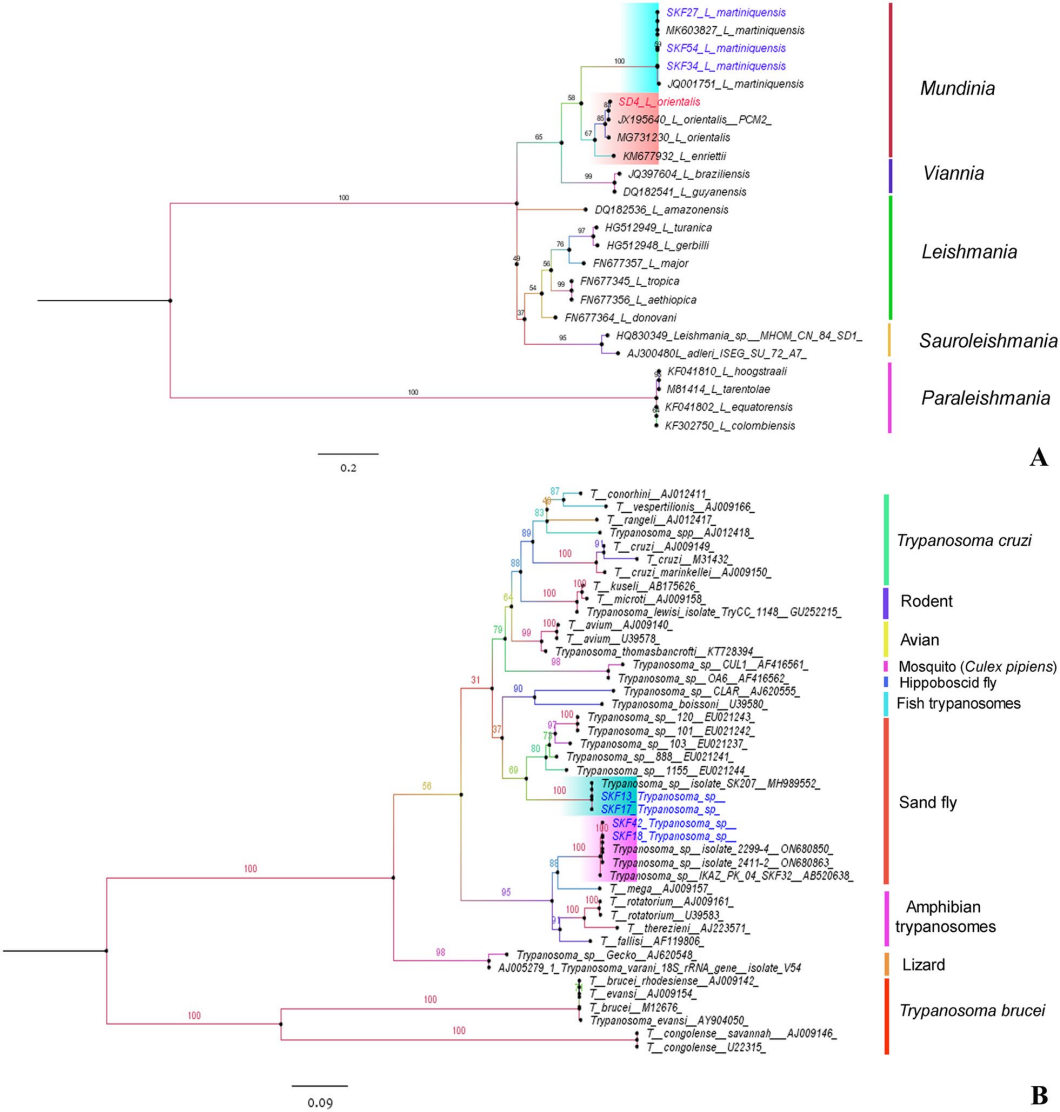
Phylogenetic trees representing the *ITS1* gene of *Leishmania* spp. **A** and *SSU rRNA* gene of *Trypanosoma* spp. **B**. These trees were constructed using IQ-TREE with maximum-likelihood bootstrap support (1000 replicates). Sequences from the Natawi and Sadao districts are distinguished by blue and red colors, respectively

All female sand flies were tested for *Leishmania* spp. and *Trypanosoma* spp. infection using *ITS1*-PCR and *SSU rRNA*-PCR, respectively. In Natawi district, three samples of *Se. khawi* tested positive for *L. martiniquensis*, while four samples of *Se. khawi* were positive for *Trypanosoma* sp. In Sadao, only one *Se. khawi* sample was positive for *L. orientalis*, showing a 99.66% identity to *L. orientalis* (isolate PCM2, accession no. JX195640) and a 99.60% identity to *L. orientalis* (isolate MHOM/TH/2021/CULE5, accession no. ON303842). The *ITS1* sequences of *Leishmania* spp. were analyzed using phylogenetic analysis alongside representative sequences of various strains and species. The findings distinctly revealed the classification of all samples into two distinct groups, *L. martiniquensis* and *L. orientalis*, within the same clade as reference sequences belonging to the *Mundinia* subgenus. These groups were notably separate from other species complexes within the subgenera *Leishmania*, *Viannia*, *Sauroleishmania*, and *Paraleishmania* (Fig. 2A). Furthermore, phylogenetic analysis of *Trypanosoma* species based on the *SSU rRNA* region demonstrated that all four sequences were distinctly classified within the *Trypanosoma* sp. isolated from sand flies in Thailand. Additionally, we observed two distinct groups of *Trypanosoma* sp. The first group was previously identified in *Se. khawi* collected from Chantaburi, Thailand (accession no. ON680850 and ON680863) and exhibited a close relationship with the amphibian trypanosome group. Meanwhile, the second group exhibited similarities to *Trypanosoma* sp. found in *Se. khawi* collected from Songkhla, Thailand (accession no. MH989552) (Fig. 2B). Interestingly, *Trypanosoma* parasites demonstrated host specificity, as evidenced by their distinct separation within the phylogenetic tree based on their respective hosts. The sequences generated in this study were deposited in the NCBI GenBank database with the following accession numbers: PP860607-PP860610 for *Trypanosoma* sp., PP862807 for *L. orientalis*, and PP862808-PP862810 for *L. martiniquensis*.

### Assessment of insecticide resistance mutations in sand flies

**Fig. 3:**
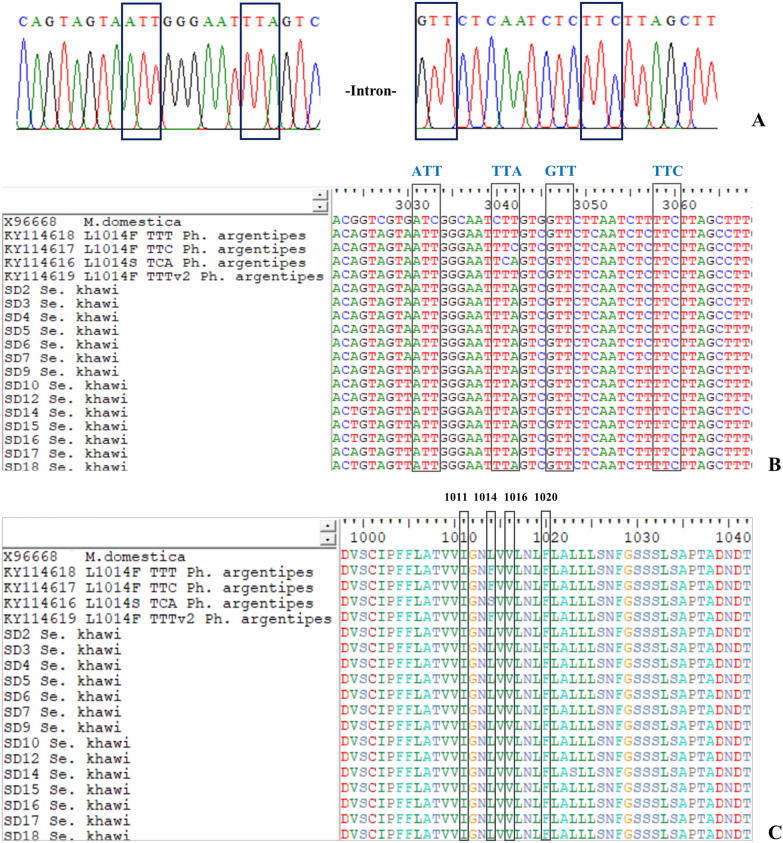
Chromatograms of homozygous genotypes demonstrating nucleotide sequencing (**A**), sequence alignment of the domain IIS6 fragment of *Vgsc* in *Sergentomyia khawi* for nucleotide sequences (**B**) and amino acid (**C**). The alignment includes the wild type of *Musca domestica* (accession number: X96668) and *Phlebotomus argentipes* (accession nos; KY114616–KY114619), highlighting amino acid positions 1011, 1014, 1016, and 1020 and nucleotide sequences indicated by a vertical column

The sequences of the 75 *Vgsc* domain IIS6 from *Se. khawi* were processed by intron removal and exon splicing to generate the translated amino acid sequence (Fig. 3A). The results revealed that all 75 samples (100%) showed no *kdr* mutation at codon 1014 with the presence of the wild-type allele (leucine, TTA). There was no replacement of leucine with serine (L1014S, TCA) or with phenylalanine (L1014F), which can occur through two alleles (TTC and TTT), compared to references sequences. Moreover, only wild-type alleles were identified at codons 1011I/I (isoleucine, ATT), 1016 V/V (valine, GTT), and 1020F/F (phenylalanine, TTC) in all samples (Fig. 3B and C).

## Discussion

Numerous cases of leishmaniasis have been reported in southern Thailand [37, 38], underlining the importance of comprehensive sand fly surveys in these areas. While previous surveys documented a variety of sand fly species, misidentification remains a significant challenge [39]. Furthermore, challenges arise from cryptic species complexes and subtle morphological differences, leading to misidentification as reported in numerous studies. Preativatanyou et al. (2023) highlighted the ambiguity between *Sergentomyia gemmea* and *Se. khawi* [40], while Phuphisut et al. (2021) provided evidence of misidentification of *Se. gemmea* as *Se. iyengari* and vice versa [41]. Additionally, Vu et al. (2021) proposed that the historical records of *Se. iyengari* in Southeast Asia may actually be relevant to *Se. khawi* [42]. The taxonomy of these species has been further confounded by the synonymization of *Se. iyengari* with *Se. hivernus* [30]. Utilizing molecular techniques that target both mitochondrial and nuclear DNA for sand fly species identification serves as a valuable and practical solution for resource conservation while confirming species identities [43, 44]. This approach enables additional molecular investigations, facilitating the generation of data on pathogen detection and identification of insecticide resistance mutations. In this study, the *CytB* gene identified sand fly species, revealing *Se. khawi* as the predominant species in both districts, with unique species distribution and dominant species in each area. Interestingly, *Sergentomyia* spp. grouped with sand flies reported in the Lao PDR [39], and specific *Se. khawi* specimens from the Sadao district formed a unique sister clade distinct from the primary *Se. khawi* clade. Rispail and Léger (1998) revealed the genus *Sergentomyia* as having the highest level of diversity among sand flies [7]. The diversity of sand fly fauna, with comparable species compositions across various environments within each area [45], suggests that the interaction between caves and their surroundings plays a significant role in sustaining sand fly communities.

In our molecular detection of pathogens in sand flies, we found *Leishmania* parasites (*L. orientalis* and *L. martiniquensis*) as well as *Trypanosoma * parasites (*Trypanosoma* sp.) in *Se. khawi*. A previous report from Thailand detected *L. martiniquensis* DNA in various sand fly species, including *Se. gammae* [46], *Se.* (*Parrotomyia*) *barraudi* [18], *Se. khawi* [19], and *Grassomyia indica* [40], all collected from the southern region. To the best of our knowledge, this study provides the first report of *L. orientalis* DNA detected in *Se. khawi* in southern Thailand. This aligns with report of an autochthonous visceral leishmaniasis case involving the *L. orientalis* strain PCM2 (formerly named *L. siamensis*) isolated from Trang province, southern Thailand [47]. These results suggest that *Se. khawi* may serve as a potential vector for *Leishmania* parasites within the *Mundinia* subgenus. However, dissections were not performed in this study to confirm the presence of metacyclic promastigotes in the sand flies. In *Se. khawi,* our analysis of *Trypanosoma*  species using the *SSU rRNA* gene identified two distinct groups of *Trypanosoma* sp. Interestingly, two samples from one group clustered closely with the amphibian trypanosome group. The previous report demonstrated that *Trypanosoma* sp. isolated from *Se. khawi* in this same area in 2018 exhibited the highest genetic differentiation, primarily being isolated from various Amazonian amphibian species [40]. However, a detection of an unknown *Trypanosoma* sp., genetically related to rodent-infecting *T. microti* and *T. kuseli*, was reported in *Ph. stantoni* collected from Songkhla province [30]. Srisuton et al. (2019) investigated that *Trypanosoma noyesi* had been identified in *Se. anodontis* and *Phlebotomus asperulus* [19]. Furthermore, sand flies from several species, including *Se. khawi*, *Gr. indica, Se. anodontis*, *Ph. asperulus*, and *Ph. betisi*, harbored an unidentified *Trypanosoma* species across all study areas. Notably, a co-infection sample of *L. martiniquensis* and *Trypanosoma* was discovered in *Se. khawi* from Songkhla Province. As aforementioned, the results indicate the ongoing circulation of *Leishmania* and *Trypanosoma* parasites in sand flies, especially *Se. khawi*, which could potentially result in future disease transmission to humans and animals.

Preventing sand fly-borne diseases relies significantly on effective vector control measures. Disease control primarily involves interrupting disease transmission by reducing the sand fly population. In Thailand, insecticide spraying is a widely used method of vector control, while pyrethroids are commonly used to target adult and immature stages of mosquitoes [31]. However, no specific sand fly control program using insecticides exists in the country. Consequently, data on sand fly insecticide resistance are not available. This study encouragingly revealed that *Se. khawi* showed no presence of known pyrethroid resistance mutations (I1011M, L1014F/S, V1016G, and F1020S) in the *Vgsc* gene. Unfortunately, due to limitations in rearing sand flies in the laboratory, we were unable to conduct bioassays to determine pyrethroid resistance phenotype. Therefore, we strongly recommend that future studies perform phenotypic analysis followed by determining the molecular mechanisms of resistance. Interestingly, a previous study reported that *Phlebotomus perfiliewi*, the primary vector of *L. infantum* in Northern Italy, showed the absence of mutations in the *Vgsc* gene, including I1011M, L1014F/S, V1016G, or F1020S [48]. Conversely, *Ph. argentipes* collected from Bangladesh showed mutant alleles (L1014F/S), but no mutations were detected at codons 1011, 1016, and 1020 [49]. Historically, sand flies have been considered generally susceptible to insecticides. However, DDT resistance in *Ph. argentipes* and *Ph. papatasi* was reported in 1979 in Bihar, India [50]. Amelia-Yap et al. (2018) revealed that over 37 resistance-associated *kdr*-type mutations or combinations of mutations have been detected in pyrethroid and DDT-resistant insect populations [51]. Recently, two *kdr* mutations at codon 1014 (L1014F and L1014S) have been investigated in sand flies in India, located in the same codon regions as described in mosquitoes [36]. L1014F is the most common *kdr* mutation in insects, whereas L1014S has only been found in mosquitoes [52]. Pathirage et al. (2020) investigated the insecticide susceptibility status of *Ph. argentipes* in Sri Lanka for the first time, examining metabolic and genetic mechanisms that may confer insecticide resistance [53]. In 2024, the *kdr* mutation L1014F and L1014S was detected in *Phlebotomus papatasi* and *Ph. tobbi*, but no *kdr* mutations were found in the *Ph. caucasicus*, *Ph. perfiliewi*, and *Ph. sergenti* in Armenia [54]. Currently, the lack of knowledge regarding the status of pyrethroid resistance in Thai sand flies hinders effective vector control. Here, we propose the first investigation of molecular markers in sand fly populations from Thailand to determine their pyrethroid resistance status using molecular genotyping assays targeting known resistance markers.

The information from this study can provide valuable insights into the prevalence of parasites in the sand fly population, the potential role of specific sand fly species as a vector in endemic areas of leishmaniasis, and insecticide resistance status of sand flies in Thailand. Nevertheless, future studies should conduct extensive surveys and collect samples from various locations across Thailand for a more comprehensive analysis.

## Conclusions

The current study indicated *Leishmania* and *Trypanosoma* parasites circulating in sand flies at Songkhla, southern Thailand. Notably, *L. orientalis* was first identified in *Se. khawi*, highlighting a potential vector for this parasite in the region. However, *kdr* mutations in *Vgsc* region were not observed in the predominant *Se. khawi*. The establishment of geo-spatial information on vectors, *Leishmania* and *Trypanosoma* parasites, and the insecticide resistance status in sand flies has the potential to significantly improve risk assessments and guide targeted vector control efforts in Thailand.

### Supplementary Information

Additional file 1: Table S1. Sampling location, species composition, and pathogen presence in sand fly populations.

## Data Availability

The datasets used and analyzed during the current study are available from the corresponding author on reasonable request. The sequence data obtained from this study have been deposited in the NCBI GenBank database (accession nos. PP860607–PP860610 for *Trypanosoma* sp., PP862807 for *Leishmania orientalis*, and PP862808-PP862810 for *L. martiniquensis*).

## References

[1] Crosskey RW. “Introduction to the Diptera.” Medical insects and arachnids. Dordrecht: Springer Netherlands, 1993. 51–77.

[2] Lane RP. “Sandflies (Phlebotominae).” Medical Insects and Arachnids. Chapman & Hall. London, 1993. 78–119.

[3] Killick-Kendrick R. The biology and control of phlebotomine sand flies. Clin Dermatol. 1999;17:279–89. 10.1016/s0738-081x(99)00046-2. 10384867 10.1016/s0738-081x(99)00046-2

[4] Akhoundi M, Kuhls K, Cannet A, Votýpka J, Marty P, Delaunay P, et al. A historical overview of the classification, evolution, and dispersion of *Leishmania* parasites and sandflies. PLoS Negl Trop Dis. 2016;10:e0004349. 10.1371/journal.pntd.0004349. 26937644 10.1371/journal.pntd.0004349 PMC4777430

[5] Maroli M, Feliciangeli MD, Bichaud L, Charrel RN, Gradoni L. Phlebotomine sandflies and the spreading of leishmaniases and other diseases of public health concern. Med Vet Entomol. 2013;27:123–47. 10.1111/j.1365-2915.2012.01034.x. 22924419 10.1111/j.1365-2915.2012.01034.x

[6] Killick-Kendrick R. Phlebotomine vectors of the leishmaniases: a review. Med Vet Entomol. 1990;4:1–24. 10.1111/j.1365-2915.1990.tb00255.x. 2132963 10.1111/j.1365-2915.1990.tb00255.x

[7] Rispail P, Léger N. Numerical taxonomy of Old World Phlebotominae (Diptera: Psychodidae). 2. Restatement of classification upon subgeneric morphological characters. Mem Inst Oswaldo Cruz. 1998;93:787–93. 10.1590/s0074-02761998000600016. 9921303 10.1590/s0074-02761998000600016

[8] Alkan C, Bichaud L, de Lamballerie X, Alten B, Gould EA, Charrel RN. Sandfly-borne phleboviruses of Eurasia and Africa: epidemiology, genetic diversity, geographic range, control measures. Antiviral Res. 2013;100:54–74. 10.1016/j.antiviral.2013.07.005. 23872312 10.1016/j.antiviral.2013.07.005

[9] World Health Organization. 2023. “Fact Sheet: Leishmaniasis.” Retrieved from www.who.int/news-room/fact-sheets/detail/leishmaniasis.

[10] Hong A, Zampieri RA, Shaw JJ, Floeter-Winter LM, Laranjeira-Silva MF. One health approach to leishmaniases: understanding the disease dynamics through diagnostic tools. Pathogens. 2020;9:809. 10.3390/pathogens9100809. 33019713 10.3390/pathogens9100809 PMC7599840

[11] Pothirat T, Tantiworawit A, Chaiwarith R, Jariyapan N, Wannasan A, Siriyasatien P, et al. First isolation of *Leishmania* from Northern Thailand: case report, identification as *Leishmania martiniquensis* and phylogenetic position within the *Leishmania enriettii* complex. PLoS Negl Trop Dis. 2014;8:e3339. 10.1371/journal.pntd.0003339. 25474647 10.1371/journal.pntd.0003339 PMC4256172

[12] Chiewchanvit S, Tovanabutra N, Jariyapan N, Bates MD, Mahanupab P, Chuamanochan M, et al. Chronic generalized fibrotic skin lesions from disseminated leishmaniasis caused by *Leishmania martiniquensis* in two patients from northern Thailand infected with HIV. Br J Dermatol. 2015;173:663–70. 10.1111/bjd.13812. 25823707 10.1111/bjd.13812

[13] Jariyapan N, Daroontum T, Jaiwong K, Chanmol W, Intakhan N, Sor-Suwan S, et al. *Leishmania* (*Mundinia*) *orientalis *n. sp. (Trypanosomatidae), a parasite from Thailand responsible for localised cutaneous leishmaniasis. Parasit Vectors. 2018;11:351. 10.1186/s13071-018-2908-3. 10.1186/s13071-018-2908-3 PMC6006788 29914526

[14] Ruang-Areerate T, Ruang-Areerate P, Manomat J, Naaglor T, Piyaraj P, Mungthin M, et al. Genetic variation and geographic distribution of *Leishmania orientalis* and *Leishmania martiniquensis* among *Leishmania*/HIV co-infection in Thailand. Sci Rep. 2023;13:23094. 10.1038/s41598-023-50604-4. 38155252 10.1038/s41598-023-50604-4 PMC10754904

[15] Thisyakorn U, Jongwutiwes S, Vanichsetakul P, Lertsapcharoen P. Visceral leishmaniasis: the first indigenous case report in Thailand. Trans R Soc Trop Med Hyg. 1999;93:23–4. 10.1016/s0035-9203(99)90166-9. 10492782 10.1016/s0035-9203(99)90166-9

[16] Maharom P, Siripattanapipong S, Mungthin M, Naaglor T, Sukkawee R, Pudkorn R, et al. Visceral leishmaniasis caused by *Leishmania infantum* in Thailand. Southeast Asian J Trop Med Public Health. 2008;39:988–90. 19062685

[17] World Health Organization. 2015. “Status of endemicity of cutaneous leishmaniasis worldwide.” Retrieved from https://apps.who.int/neglected_diseases/ntddata/leishmaniasis/leishmaniasis.html.

[18] Chusri S, Thammapalo S, Silpapojakul K, Siriyasatien P. Animal reservoirs and potential vectors of *Leishmania siamensis* in southern Thailand. Southeast Asian J Trop Med Public Health. 2014;45:13–9. 24964648

[19] Srisuton P, Phumee A, Sunantaraporn S, Boonserm R, Sor-Suwan S, Brownell N, et al. Detection of *Leishmania* and *Trypanosoma* DNA in field-caught sand flies from endemic and non-endemic areas of leishmaniasis in Southern Thailand. Insects. 2019;10:238. 10.3390/insects10080238. 31382501 10.3390/insects10080238 PMC6722825

[20] Lee YF, Cheng CC, Lin NN, Liu SA, Tung KC, Chiu YT. Isolation of *Trypanosoma* (*Megatrypanum*) *theileri* from dairy cattle in Taiwan. J Vet Med Sci. 2010;72:417–24. 10.1292/jvms.09-0343. 20009352 10.1292/jvms.09-0343

[21] Jittapalapong S, Inpankaew T, Sarataphan N, Herbreteau V, Hugot JP, Morand S, et al. Molecular detection of divergent trypanosomes among rodents of Thailand. Infect Genet Evol. 2008;8:445–9. 10.1016/j.meegid.2007.08.002. 17904918 10.1016/j.meegid.2007.08.002

[22] Tang HJ, Lan YG, Wen YZ, Zhang XC, Desquesnes M, Yang TB, et al. Detection of *Trypanosoma lewisi* from wild rats in Southern China and its genetic diversity based on the *ITS1* and *ITS2* sequences. Infect Genet Evol. 2012;12:1046–51. 10.1016/j.meegid.2012.02.018. 22449774 10.1016/j.meegid.2012.02.018

[23] Hatama S, Shibahara T, Suzuki M, Kadota K, Uchida I, Kanno T. Isolation of a Megatrypanum trypanosome from sika deer (*Cervus nippon yesoensis*) in Japan. Vet Parasitol. 2007;149:56–64. 10.1016/j.vetpar.2007.07.019. 17714873 10.1016/j.vetpar.2007.07.019

[24] Sarataphan N, Vongpakorn M, Nuansrichay B, Autarkool N, Keowkarnkah T, Rodtian P, et al. Diagnosis of a *Trypanosoma lewisi*-like (Herpetosoma) infection in a sick infant from Thailand. J Med Microbiol. 2007;56:1118–21. 10.1099/jmm.0.47222-0. 17644723 10.1099/jmm.0.47222-0 PMC3066167

[25] Luckins AG. *Trypanosoma evansi* in Asia. Parasitol Today. 1988;4:137–42. 10.1016/0169-4758(88)90188-3. 15463067 10.1016/0169-4758(88)90188-3

[26] Hamilton PB, Gibson WC, Stevens JR. Patterns of co-evolution between trypanosomes and their hosts deduced from ribosomal RNA and protein-coding gene phylogenies. Mol Phylogenet Evol. 2007;44:15–25. 10.1016/j.ympev.2007.03.023. 17513135 10.1016/j.ympev.2007.03.023

[27] Zeledón R, Rosabal R. *Trypanosoma leonidasdeanei* sp. nov. in insectivorous bats of Costa Rica. Ann Trop Med Parasitol. 1969 Jun;63:221–8. 10.1080/00034983.1969. 10.1080/00034983.1969.11686623 5392759

[28] Viola LB, Campaner M, Takata CS, Ferreira RC, Rodrigues AC, Freitas RA, et al. Phylogeny of snake trypanosomes inferred by *SSU rDNA* sequences, their possible transmission by phlebotomines, and taxonomic appraisal by molecular, cross-infection and morphological analysis. Parasitology. 2008;135:595–605. 10.1017/S0031182008004253. 18371240 10.1017/S0031182008004253

[29] Gramiccia M, Gradoni L, Maroli M. Isoenzyme characterization of *Trypanosoma platydactyli catouillard* 1909 isolated from *Sergentomyia minuta minuta* (Rondani 1843) in Italy. Ann Parasitol Hum Comp. 1989;1989:154–6. 10.1051/parasite/1989642154.

[30] Phumee A, Tawatsin A, Thavara U, Pengsakul T, Thammapalo S, Depaquit J, et al. Detection of an unknown *Trypanosoma* DNA in a *Phlebotomus stantoni* (Diptera: Psychodidae) collected from southern Thailand and records of new sand flies with reinstatement of *Sergentomyia hivernus* Raynal & Gaschen, 1935 (Diptera: Psychodidae). J Med Entomol. 2017;54:429–34. 10.1093/jme/tjw161. 27744363 10.1093/jme/tjw161

[31] Chareonviriyaphap T, Bangs MJ, Suwonkerd W, Kongmee M, Corbel V, Ngoen-Klan R. Review of insecticide resistance and behavioral avoidance of vectors of human diseases in Thailand. Parasit Vectors. 2013;6:280. 10.1186/1756-3305-6-280. 24294938 10.1186/1756-3305-6-280 PMC3850650

[32] Davies TG, Field LM, Usherwood PN, Williamson MS. A comparative study of voltage-gated sodium channels in the Insecta: implications for pyrethroid resistance in Anopheline and other Neopteran species. Insect Mol Biol. 2007;16:361–75. 10.1111/j.1365-2583.2007.00733.x. 17433068 10.1111/j.1365-2583.2007.00733.x

[33] Ready PD, Day JC, de Souza AA, Rangel EF, Davies CR. Mitochondrial DNA characterization of populations of *Lutzomyia whitmani* (Diptera: Psychodidae) incriminated in the peri-domestic and silvatic transmission of *Leishmania* species in Brazil. Bull Entom Res. 1997;87:187–95. 10.1017/S0007485300027346.

[34] Spanakos G, Piperaki ET, Menounos PG, Tegos N, Flemetakis A, Vakalis NC. Detection and species identification of Old World *Leishmania* in clinical samples using a PCR-based method. Trans R Soc Trop Med Hyg. 2008;102:46–53. 10.1016/j.trstmh.2007.05.019. 17669452 10.1016/j.trstmh.2007.05.019

[35] Noyes HA, Stevens JR, Teixeira M, Phelan J, Holz P. A nested PCR for the ssrRNA gene detects *Trypanosoma binneyi* in the platypus and *Trypanosoma* sp. in wombats and kangaroos in Australia. Int J Parasitol. 1999;29:331–9. 10.1016/s0020-7519(98)00167-2. 10221634 10.1016/s0020-7519(98)00167-2

[36] Gomes B, Purkait B, Deb RM, Rama A, Singh RP, Foster GM, et al. Knockdown resistance mutations predict DDT resistance and pyrethroid tolerance in the visceral leishmaniasis vector *Phlebotomus argentipes*. PLoS Negl Trop Dis. 2017;11:e0005504. 10.1371/journal.pntd.0005504. 28414744 10.1371/journal.pntd.0005504 PMC5407848

[37] Songumpai N, Promrangsee C, Noopetch P, Siriyasatien P, Preativatanyou K. First evidence of co-circulation of emerging *Leishmania martiniquensis*, *Leishmania orientalis*, and *Crithidia* sp. in *Culicoides* biting midges (Diptera: Ceratopogonidae), the putative vectors for autochthonous transmission in southern Thailand. Trop Med Infect Dis. 2022;7:379. 10.3390/tropicalmed7110379. 36422930 10.3390/tropicalmed7110379 PMC9696774

[38] Leelayoova S, Siripattanapipong S, Manomat J, Piyaraj P, Tan-Ariya P, Bualert L, et al. Leishmaniasis in Thailand: a review of causative agents and situations. Am J Trop Med Hyg. 2017;96:534–42. 10.4269/ajtmh.16-0604. 28093539 10.4269/ajtmh.16-0604 PMC5361524

[39] Depaquit J, Vongphayloth K, Siriyasatien P, Polseela R, Phumee A, Loyer M, et al. On the true identity of *Sergentomyia gemmea* and description of a closely related species: *Se. raynali* n. sp. Med Vet Entomol. 2019;33:521–9. 10.1111/mve.12393. 31155766 10.1111/mve.12393

[40] Preativatanyou K, Chinwirunsirisup K, Phumee A, Khositharattanakool P, Sunantaraporn S, Depaquit J, et al. Species diversity of phlebotomine sand flies and sympatric occurrence of *Leishmania* (*Mundinia*) *martiniquensis*, *Leishmania* (*Leishmania*) *donovani* complex, and *Trypanosoma* spp. in the visceral leishmaniasis focus of southern Thailand. Acta Trop. 2023;244:106949. 10.1016/j.actatropica.2023.106949. 37211153 10.1016/j.actatropica.2023.106949

[41] Phuphisut O, Nitatsukprasert C, Pathawong N, Jaichapor B, Pongsiri A, Adisakwattana P, et al. Sand fly identification and screening for *Leishmania* spp. in six provinces of Thailand. Parasit Vectors. 2021;14:352. 10.1186/s13071-021-04856-6. 34217359 10.1186/s13071-021-04856-6 PMC8254935

[42] Vu SN, Tran HS, Tran VP, Tran CT, Tran ND, Dang DA, et al. Taxonomical insights and ecology of sandfly (Diptera, Psychodidae) species in six provinces of Northern Vietnam. Parasite. 2021;28:85. 10.1051/parasite/2021080. 34928207 10.1051/parasite/2021080 PMC8686828

[43] Aransay AM, Scoulica E, Tselentis Y, Ready PD. Phylogenetic relationships of phlebotomine sandflies inferred from small subunit nuclear ribosomal DNA. Insect Mol Biol. 2000;9:157–68. 10.1046/j.1365-2583.2000.00168.x. 10762423 10.1046/j.1365-2583.2000.00168.x

[44] Contreras Gutiérrez MA, Vivero RJ, Vélez ID, Porter CH, Uribe S. DNA barcoding for the identification of sand fly species (Diptera, Psychodidae, Phlebotominae) in Colombia. PLoS ONE. 2014;9:e85496. 10.1371/journal.pone.0085496. 24454877 10.1371/journal.pone.0085496 PMC3893204

[45] Campos AM, Maia RDA, Capucci D, Paglia AP, Andrade Filho JD. Species composition of sand flies (Diptera: Psychodidae) in caves of Quadrilátero Ferrífero, state of Minas Gerais, Brazil. PLoS ONE. 2020;15:e0220268. 10.1371/journal.pone.0220268. 32155153 10.1371/journal.pone.0220268 PMC7064241

[46] Kanjanopas K, Siripattanapipong S, Ninsaeng U, Hitakarun A, Jitkaew S, Kaewtaphaya P, et al. *Sergentomyia* (*Neophlebotomus*) *gemmea*, a potential vector of *Leishmania siamensis* in southern Thailand. BMC Infect Dis. 2013;13:333. 10.1186/1471-2334-13-333. 23870062 10.1186/1471-2334-13-333 PMC3725172

[47] Leelayoova S, Siripattanapipong S, Hitakarun A, Kato H, Tan-ariya P, Siriyasatien P, et al. Multilocus characterization and phylogenetic analysis of *Leishmania siamensis* isolated from autochthonous visceral leishmaniasis cases, southern Thailand. BMC Microbiol. 2013;13:60. 10.1186/1471-2180-13-60. 23506297 10.1186/1471-2180-13-60 PMC3724499

[48] Balaska S, Calzolari M, Grisendi A, Scremin M, Dottori M, Mavridis K, et al. Monitoring of insecticide resistance mutations and pathogen circulation in sand flies from Emilia-Romagna, a leishmaniasis endemic region of Northern Italy. Viruses. 2023;15:148. 10.3390/v15010148. 36680189 10.3390/v15010148 PMC9862798

[49] Sarkar SR, Kuroki A, Özbel Y, Osada Y, Omachi S, Shyamal PK, et al. First detection of voltage-gated sodium channel mutations in *Phlebotomus argentipes* collected from Bangladesh. J Vector Borne Dis. 2021;58:368–73. 10.4103/0972-9062.328972. 35381827 10.4103/0972-9062.328972

[50] Mukhopadhyay AK, Saxena NB, Narasimham MV. Susceptibility status of *Phlebotomus argentipes* to DDT in some kala-azar endemic areas of Bihar (India). Indian J Med Res. 1990;91:458–60. 2091993

[51] Amelia-Yap ZH, Chen CD, Sofian-Azirun M, Low VL. Pyrethroid resistance in the dengue vector *Aedes aegypti* in Southeast Asia: present situation and prospects for management. Parasit Vectors. 2018;11:332. 10.1186/s13071-018-2899-0. 29866193 10.1186/s13071-018-2899-0 PMC5987412

[52] Rinkevich FD, Du Y, Dong K. Diversity and convergence of sodium channel mutations involved in resistance to pyrethroids. Pestic Biochem Physiol. 2013;106:93–100. 10.1016/j.pestbp.2013.02.007. 24019556 10.1016/j.pestbp.2013.02.007 PMC3765034

[53] Pathirage DRK, Karunaratne SHPP, Senanayake SC, Karunaweera ND. Insecticide susceptibility of the sand fly leishmaniasis vector *Phlebotomus argentipes* in Sri Lanka. Parasit Vectors. 2020;13:246. 10.1186/s13071-020-04117-y. 32404115 10.1186/s13071-020-04117-y PMC7218544

[54] Paronyan L, Babayan L, Vardanyan H, Manucharyan A, Papapostolou KM, Balaska S, et al. Molecular monitoring of insecticide resistance in major disease vectors in Armenia. Parasit Vectors. 2024;17:54. 10.1186/s13071-024-06139-2. 38321481 10.1186/s13071-024-06139-2 PMC10848433

